# Natural killer cells affect the natural course, drug resistance, and prognosis of multiple myeloma

**DOI:** 10.3389/fcell.2024.1359084

**Published:** 2024-02-12

**Authors:** Li Zhang, Xiaohuan Peng, Tao Ma, Jia Liu, Zhigang Yi, Jun Bai, Yanhong Li, Lijuan Li, Liansheng Zhang

**Affiliations:** ^1^ Department of Hematology, Lanzhou University Second Hospital, Lanzhou University, Lanzhou, China; ^2^ Key Laboratory of the Hematology of Gansu Province, Lanzhou University Second Hospital, Lanzhou University, Lanzhou, China; ^3^ Department of Hematology, The Affiliated Hospital of Southwest Medical University, Luzhou, China

**Keywords:** multiple myeloma, NK cells, proteasome inhibitors, immunomodulatory drugs, monoclonal antibodies, autologous hematopoietic stem cell transplantation, chimeric antigen receptor cells

## Abstract

Multiple myeloma (MM), a stage-developed plasma cell malignancy, evolves from monoclonal gammopathy of undetermined significance (MGUS) or smoldering MM (SMM). Emerging therapies including immunomodulatory drugs, proteasome inhibitors, monoclonal antibodies, chimeric antigen-T/natural killer (NK) cells, bispecific T-cell engagers, selective inhibitors of nuclear export, and small-molecule targeted therapy have considerably improved patient survival. However, MM remains incurable owing to inevitable drug resistance and post-relapse rapid progression. NK cells with germline-encoded receptors are involved in the natural evolution of MGUS/SMM to active MM. NK cells actively recognize aberrant plasma cells undergoing malignant transformation but are yet to proliferate during the elimination phase, a process that has not been revealed in the immune editing theory. They are potential effector cells that have been neglected in the therapeutic process. Herein, we characterized changes in NK cells regarding disease evolution and elucidated its role in the early clinical monitoring of MM. Additionally, we systematically explored dynamic changes in NK cells from treated patients who are in remission or relapse to explore future combination therapy strategies to overcome drug resistance.

## 1 Introduction

Multiple myeloma (MM), the third most common hematologic malignancy, is characterized by the clonal proliferation of plasma cells, bone injury, anemia, renal failure, and hypercalcemia (Sung et al., 2021). Active MM frequently emerges as a result of the progression of monoclonal gammopathy of undetermined significance (MGUS) and smoldering multiple myeloma (SMM), with approximately 1% of patients with MGUS and 10% of patients with SMM progressing to a clinical stage requiring treatment each year. Current therapeutic advances, including immunomodulatory drugs (IMiDs), proteasome inhibitors (PIs), and anti-CD38 antibodies, have markedly improved the outcomes of patients with newly diagnosed MM (NDMM). However, MM remains incurable; an effective approach to optimizing patient survival is to stop progression from the precancerous state to the active stage.

**FIGURE 1:**
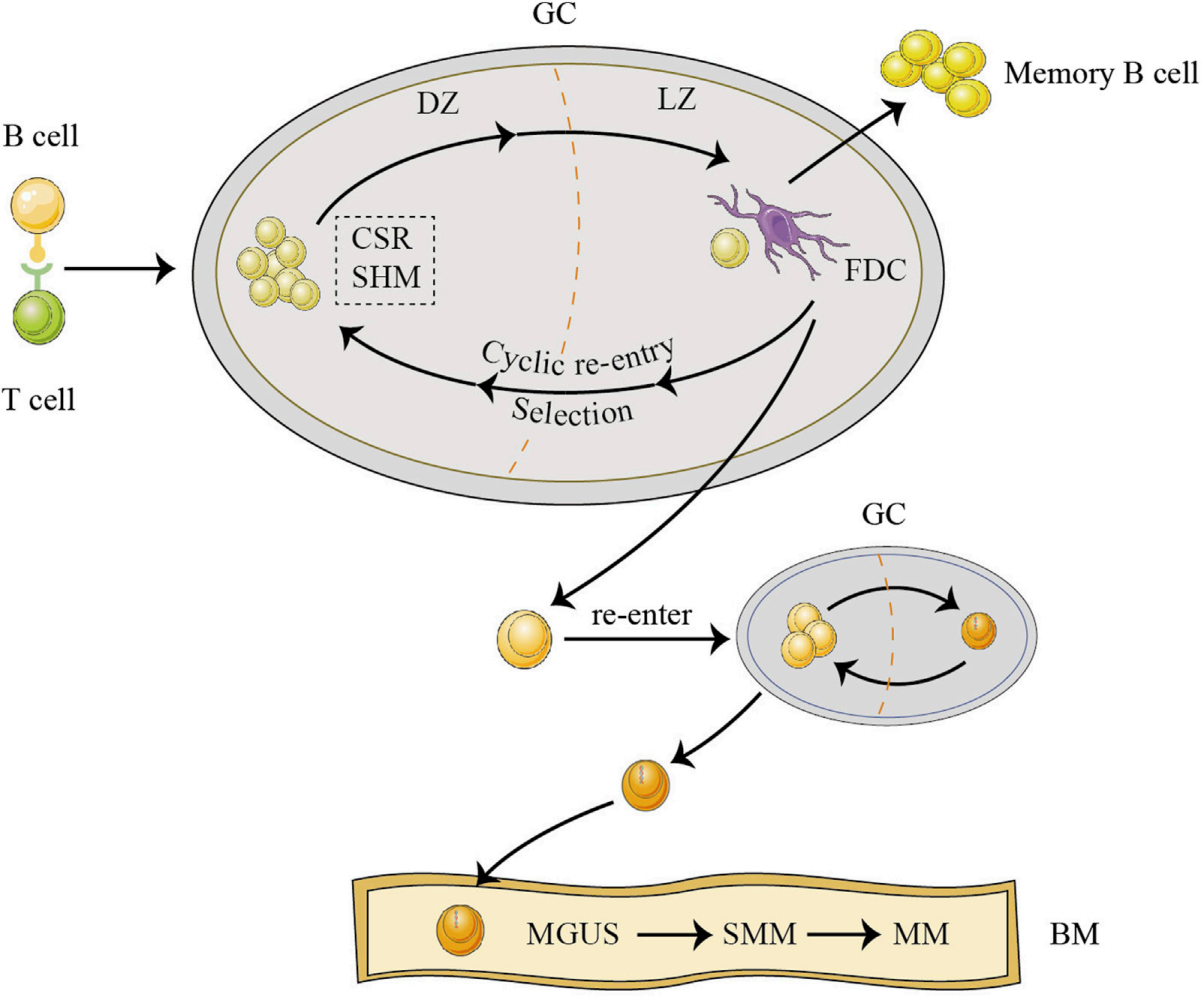
The origins of multiple myeloma (MM). Before developing MM, abnormal cells in the germinal center (GC) endure a lengthy pro-monoclonal gammopathy of undetermined significance (MGUS) phase. B cells originating from GC proliferate rapidly in the dark zone (DZ), undergo class switch recombination (CSR) and somatic hypermutation (SHM), and are subsequently selected by follicular dendritic cell (FDC) in the light zone (LZ), re-entering the DZ to undergo repeated cyclic re-entry processes (Basso, 2021; Pasqualucci and Klein, 2022). After accumulating generation by generation, a clone that has acquired a critical mutation leaves the GC and, subsequently, re-enters the GC to acquire the initiating mutation (Maura et al., 2021; Ho et al., 2022). Finally, the clone, independent of the GC, moves to the bone marrow (BM) by chemokines and then begins to evolve from MGUS to smoldering MM (SMM) to active MM.

Myeloma arises from B lymphocytes located in the germinal centers (GC) of the lymph nodes. The GC contains the dark zone (DZ) and light zone (LZ). In the DZ, antigen-stimulated B and T cells recognize each other, rapidly proliferate, and generate huge amounts antibodies through class switch recombination (CSR) and somatic hypermutation (SHM) (Basso, 2021). Activated cytidine deaminase coordinates this process. Subsequently, such B lymphocytes either bind with follicular dendritic cells (FDCs) in the LZ or become apoptotic cells (Basso, 2021). B cells selected by FDCs continue to undergo repeated CSR and SHM between the DZ and LZ, a process called “cyclic re-entry.” Eventually, these cells leave the GC as memory B cells or long-lived plasma cells (Pasqualucci and Klein, 2022). Among the abovementioned processes, B cells exhibit hyperdiploidy if there are errors in chromosome segregation during rapid proliferation, CSR is susceptible to IgH translocations, and SHM is mainly in the form of single base substitutions. As a result, these three mutation types are common initiating mutations in MM (Ho et al., 2022). During the process of “cyclic re-entry,” genetic mutations accumulate from generation to generation; eventually, a clone that has acquired a critical mutation leaves the GC and re-enters the GC to acquire the initiating mutation. This is the long pre-MGUS phase (Maura et al., 2021). Finally, the clone migrates to the bone marrow (BM) independently of the GC in the presence of chemokines, where it evolves from MGUS to SMM and then to MM (Figure 1). MGUS had identical copy number aberrations and somatic mutations as MM, although with a lower frequency (Gonsalves and Rajkumar, 2022). MM progression proceeds in a branching rather than in a linear manner, leading to substantial clonal diversity and coexistence of wide genetic heterogeneity (Gonsalves and Rajkumar, 2022). Some patients with MGUS who exhibit persistent clinical inertia carry driver mutations and intraclonal evolution in MM. Moreover, subclones with potentially high-risk lesions do not become the dominant lesions in the MGUS phase (Dhodapkar, 2016). Therefore, the choice of B cells to differentiate into malignant or normal cells depends on the accumulation of genetic mutations. The higher significance of genetic alterations possibly lies in the ultimate risk assessment of malignant transformation. The reciprocal remodeling occurring between myeloma cells and immune cells plays a crucial role in regulating the process of malignant transformation. This remodeling contributes to clonal selection and creates the microenvironment that facilitates the transition from MGUS to MM. In the “immune editing” theory of cancer, MGUS and SMM are stages of immune homeostasis, suggesting the presence of an immune elimination phase before these stages. Myeloma exhibits an increase in the expression of HLA class I molecules during the process of transitioning from MGUS to MM (Bernal et al., 2009). This upregulation is associated with target cell recognition by germline-encoded receptors on the NK cell surface. Based on the reciprocity of immune editing, abnormal plasma cells may trigger the recognition of NK cells during the early immune elimination phase. Focusing on NK cells can help identify plasma cells that have achieved “malignant transformation” but have not undergone major clonal proliferation as well as determine precancerous lesions that may require early intervention.

## 2 NK cells change parallel to myeloma progression

With MM progression, NK cells exhibit changes in number and phenotype. NK cells are generally increased or unchanged in the peripheral blood in early disease stages but begin to decline in the advanced stages (Khan et al., 2019; Ho et al., 2022). Based on the present study, immune editing between NK and tumor cells may occur in the early stages and throughout disease evolution. As the disease progresses from the MGUS stage, plasma cells in patients upregulate MHC class I expression to evade NK cell recognition and overexpress Erp5 to promote MHC class I polypeptide-related sequence A (MICA) shedding into soluble MICA to induce the functional inhibition of NKG2D, an activating receptor (Vulpis et al., 2022). Subsequently, anti-MICA antibodies are present in high titers during the MGUS phase (Schneiderova et al., 2017). The degranulation level of CD56^low^CD16^low^NK cells is already impaired in patients at this stage (Vulpis et al., 2018). Moreover, the heterogeneity of cytotoxic cells at the SMM stage is associated with the advancement of the disease and the effectiveness of treatment for patients with HR SMM. The first phase is dominated by abundant NK cell numbers and depleted CD8^+^ T cell numbers, reflecting the innate or transitional immune environment. In the second phase, activated cytotoxic T cells become abundant, with a decrease in tumor load. In the third stage, the immune microenvironment is characterized by the widespread suppression and inactivation of cytotoxic cells and disease progression (Fernandez et al., 2022). Another study has reported similar alterations (Isola et al., 2021). Patients with HR SMM is not only characterized by the enrichment of gene sets associated with cytotoxic responses, including Tbet, perforin, granzyme b (GZMB), and granulysin, but also by the overexpression of suppressor molecules such as LAG-3, TIGIT, and IDO1 (17). Therefore, during the SMM stage, antitumor immune responses are activated while the immunosuppressive microenvironment is actively constructed. During the follow-up of patients with HR SMM progressing from an “asymptomatic” state to a stage requiring clinical treatment, only NK cells underwent remarkable changes. The absolute number of CD158a^+^CD56^dim^ NK cells decreased to half of that in the asymptomatic stage, with downregulation of CD16 in CD56^bright^ NK cells (Paiva et al., 2016). Furthermore, patients with HR SMM who responded to combination therapy with lenalidomide and dexamethasone primarily exhibited phenotypic changes in CD56^dim^ NK cells, for example, downregulation of CD158a and killer cell inhibitory receptors (KIRs) (Paiva et al., 2016).

**FIGURE 2:**
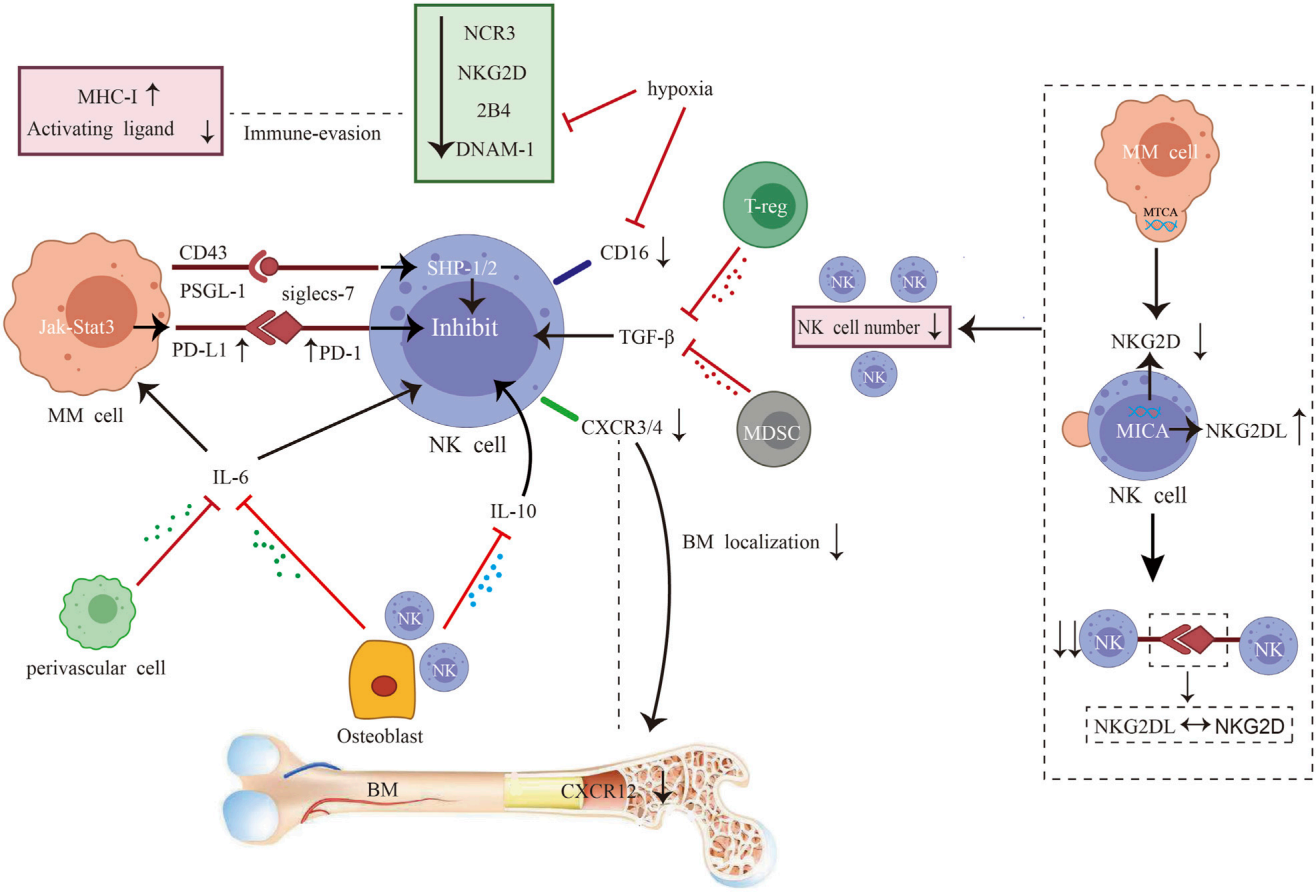
Interplay between myeloma cells and natural killer (NK) cells. After progression to active multiple myeloma (MM), NK cells are progressively depleted, with a decrease in numbers, inhibitory and activating receptor imbalance, functional inhibition, and chemokine imbalance. The combination of impaired NK cell proliferation and NKG2D-NKG2DL axis-induced fratricide led to decreased cell numbers (Seymour et al., 2022). The levels of NK cell activating receptors NCR3, NKG2D, 2B4, and DNAM-1 are reduced, while inhibiting receptor PD-1 are increased (Seymour et al., 2022). On the target cells, the inhibitory mediators MHC I and PD-L1 are also upregulated. Severe imbalance of activating and inhibiting receptors leads to functional inhibition. This alteration is associated with cytokines and hypoxia. Physical contact between osteoblasts and NK cells increases interleukin (IL)-6 and IL-10 production (Uhl et al., 2022). Regulatory T cells (Tregs) and bone marrow (BM)-derived suppressor cells release TGF-β (Ghiringhelli et al., 2005). This results in the formation of an extensive immunosuppressive microenvironment. Hypoxia decreases NKG2D and CD16 expression and impairs NK cell degranulation (Sarkar et al., 2013). Sialic acid-binding immunoglobulin-like lectin (Siglec) ligand (PSGL-1/CD43) on MM cells binds to inhibitory Siglec-7 on NK cells, inhibiting cytotoxicity and cytokine production by activating the phosphatase SHP-1/2 in NK cells (Daly et al., 2022a; Daly et al., 2022b). Downregulation of C-X-C motif chemokine (CXCL)12 and its ligand C-X-C chemokine receptor type 4 (CXCR4) affects NK cell trafficking in the BM and weakens antitumor immune responses at the primary tumor site (Ponzetta et al., 2015; Tomaipitinca et al., 2021).

After progression to active MM, NK cells are progressively depleted, with a decrease in numbers, inhibitory and activating receptor imbalance, functional inhibition, and chemokine imbalance (Figure 2). Preclinical studies have shown that myeloma cells release microvesicles comprising MICA-related genes. Such microvesicles induce the downregulation of NKG2D and transfer of NKG2DL to the surface of cells after internalization by NK cells. Subsequently, the NKG2D–NKG2DL axis facilitates NK cell fratricide (Vulpis et al., 2022). Moreover, there is a significant decrease in the NK cell activating receptors NCR3, NKG2D, 2B4, and DNAM-1 and upregulation of the inhibitory receptor PD-1 in patients (Seymour et al., 2022). The inhibitory ligands MHC I and PD-L1 are upregulated in target cells. Severe imbalance of activating and inhibiting receptors leads to functional inhibition. This alteration is associated with cytokines and hypoxia. *In vitro* preclinical studies have shown that physical contact between osteoblasts and NK cells increases interleukin (IL)-6 and IL-10 production (Uhl et al., 2022). Regulatory T cells (Tregs) and BM-derived suppressor cells release TGF-β (Ghiringhelli et al., 2005). This results in the formation of an extensive immunosuppressive microenvironment. Hypoxia decreases NKG2D and CD16 expression and impairs NK cell degranulation in preclinical studies (Sarkar et al., 2013). Furthermore, preclinical studies have shown that the sialic acid-binding immunoglobulin-like lectin (Siglec) ligand (PSGL-1/CD43) on MM cells binds to inhibitory Siglec-7 on NK cells, inhibiting cytotoxicity and cytokine production by activating SHP-1/2 in NK cells *in vitro* (Daly et al., 2022a). Downregulation of the chemokine C-X-C motif chemokine (CXCL)12 and its ligand C-X-C chemokine receptor type 4 (CXCR4) affects NK cell trafficking in the BM, and weakens antitumor immune responses at the primary tumor site in patients (Tomaipitinca et al., 2021) (Figure 2).

Studies on stage-related NK cells after progression to the clinical stage are lacking. Similar to the heterogeneity within SMM, myeloma cells possibly induce specific NK cells that can overcome the inhibitory microenvironment to exert antitumor effects within different stages. Activation or exogenous infusion of such cells could be a promising therapeutic approach. In summary, NK cells most likely recognize early abnormal plasma cells and participate in the entire process of immune editing (i.e., elimination, homeostasis, and escape) in MM. NK cells screen for clonal subpopulations resistant to innate immune attack by flexibly altering the autoimmune phenotype and subpopulation ratios. This clonal subset is often dormant at the primary tumor site and is looking for an opportunity to recur in a highly immunosuppressive microenvironment. Disease progression to the active stage could potentially be prevented at an early stage by elucidating the changes in NK cells during disease progression. In addition to the changes that occur during the natural course of the disease, treated tumors also experience functional dormancy [complete remission (CR)] and tumor proliferation (disease recurrence) associated with NK cells. Such changes are often associated with the strike or activation of the tumor microenvironment by the treatment strategy.

## 3 Involvement of NK cells in the anti-tumor response of existing therapies for MM

Currently available therapies for MM directly or indirectly influence NK cells to exert antitumor activity and result in various adaptive changes in prognosis-related NK cells after the intervention. The main therapies include the use of PIs, IMiDs, monoclonal antibodies (mAbs), autologous hematopoietic stem cell transplantation (auto-HSCT), chimeric antigen receptor (CAR) cells, bispecific antibodies (BsAbs) or trispecific antibodies, dendritic cell (DC) vaccination, histone deacetylase inhibitors (HDACis), selinexor, and venetoclax.

### 3.1 Changes in NK cell and potential applications during PI treatment

**FIGURE 3:**
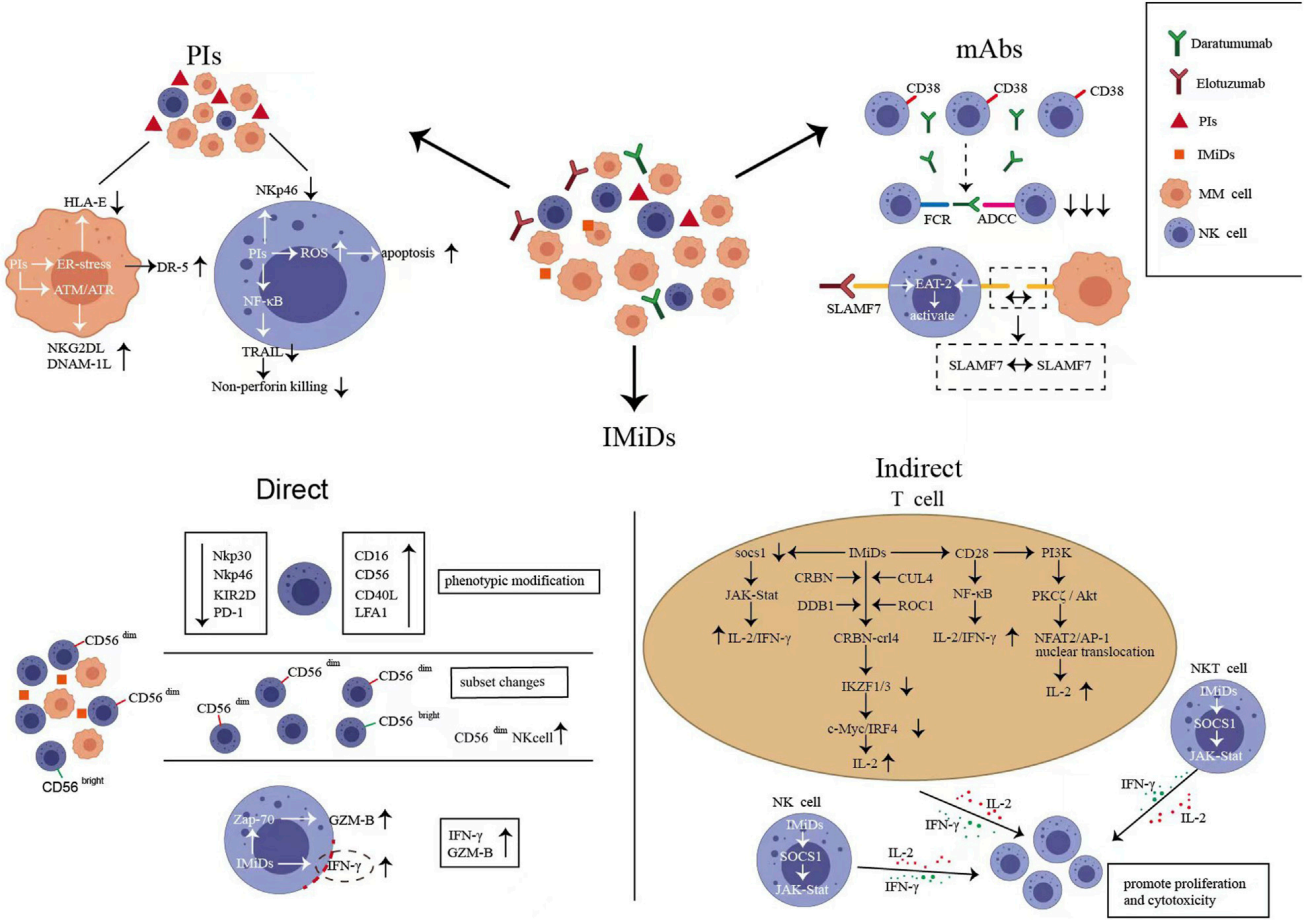
Natural killer (NK) cell changes after proteasome inhibitors (PIs), immunomodulatory drugs (IMiDs) and monoclonal antibodies (mAbs) treatment. **(A)**. Bortezomib can up- or downregulate the expression of ligands associated with NK cell activation sensitizing myeloma cells to recognize NK cells (Soriani et al., 2009; Yang et al., 2015; Niu et al., 2017), meanwhile, bortezomib inhibits the responses of NK cells to sensitized tumor cells by inducing apoptosis, decreasing activating ligand expression, and inhibiting non-perforin killing (Wang et al., 2009). **(B)**. Directly, lenalidomide alters NK cell immune phenotype and adjusts the ratio of CD56bright/dim NK cell subsets; lenalidomide activates Zap-70 in NK cells to upregulate GZM-B and increases the porous region of the actin-network to promote the release of interferon (IFN)-γ-containing vesicles (Giuliani et al., 2017; Hideshima et al., 2021); indirectly, lenalidomide promotes the proliferation and activation of NK cells by regulating the complex signaling pathways of effector cells such as T cells, NK cells, and natural killer T (NKT) cells to promote IL-2 and IFN-γ release (LeBlanc et al., 2004; Zhu et al., 2019). **(C)**. Daratumumab induced CD38^+^ NK cell fratricide via the antibody-dependent cell-mediated cytotoxicity effects of NK–NK cells (Wang et al., 2018). **(D)**. elotuzumab activates NK cells by directly binding to SLAMF7; SLAMF7-SLAMF7 interaction between NK cells and myeloma cells induced NK cell activation and promoted cytotoxicity (Malaer and Mathew, 2017).

Bortezomib exerts anti-myeloma effects by directly inducing MM cell apoptosis and inhibiting NF-κB activation and adhesion to BM stromal cells. However, this mechanism of PIs sensitizing MM cells to the recognition of NK cells remains unexplored. HLA class I on myeloma cells often leads to the inhibition of NK cell activity. PIs downregulate HLA class I molecules to induce a “self-deficient” state to activate NK cells (Yang et al., 2015). PI-treated myeloma cell lines have suppressed expression of HLA-E and are more easily targeted by NKG2A^+^ NK cells (Carlsten et al., 2019). Furthermore, bortezomib upregulates NKG2D in NK cells and DNAM-1-related ligands in myeloma cells by activating ataxia-templated mutation and RAD3-related protein (ATR)-dependent senescence program (Soriani et al., 2009; Niu et al., 2017). Endoplasmic reticulum (ER) stress induced by Bortezomib activates the unfolded protein response. This aids myeloma cells in avoiding apoptosis and developing tolerance to drugs. This mechanism results in the exposure of calreticulin and the upregulation of DR5 in myeloma cells (Zitvogel and Kroemer, 2021). Recent studies have shown that NK cells eliminate ER-stressed cells by recognizing calreticulin via NKp46 (Sen Santara et al., 2023). The upregulation of DR5 enhances tumor necrosis factor-related apoptosis-inducing ligand (TRAIL)-mediated NK cytotoxicity (Carlsten et al., 2019). Taken together, bortezomib sensitizes myeloma cells to NK cell recognition by up- or downregulate expression of ligands associated with NK cell activation (Figure 3). However, effector cells that work well against sensitizing myeloma cells may be absent in patients. *In vitro* studies have reported that bortezomib affects the antitumor capacity of NK cells via the following mechanisms: inducing the apoptosis of quiescent NK cells via the reactive oxygen species-dependent pathway; decreasing the activating receptor NKp46 (Wang et al., 2009); and downregulating TRAIL by inhibiting the NK-κB pathway, which decreases the apoptosis of target cells and significantly inhibits nonperforin killing (Feng et al., 2010). Furthermore, previous studies have reported that at clinically relevant concentrations (10 nM), bortezomib does not affect the function of NK cells (Shi et al., 2008). However, another *in vitro* study has reported that primary quiescent NK cells are sensitive to bortezomib-induced apoptosis at a concentration of 12.2 nM (Wang et al., 2009). During bortezomib treatment, the proportion of circulating NK cells decreases significantly (Kakoo et al., 2021). The role of such changes in driving clinical infections, including herpesvirus reactivation, cannot be excluded. In conclusion, bortezomib inhibits the responses of NK cells to sensitized tumor cells by inducing apoptosis, decreasing activating ligand expression, and inhibiting non-perforin killing (Figure 3). The immunosurveillance function of NK cells may be impacted by the negative regulatory effect of bortezomib on them. Therefore, this negative regulatory effect should be considered when exploring NK cell-based combination therapies. In addition, co-infusion of NK cell donor lymphocytes after bortezomib therapy may be an effective strategy to eradicate bortezomib-escaped myeloma cells, facilitating deeper therapeutic remission and delaying disease recurrence.

### 3.2 IMiDs positively regulate the immunosurveillance of NK cells

IMiDs exert anti-myeloma effects via immunomodulatory, antiangiogenic, anti-inflammatory, and antiproliferative mechanisms. The co-activating effect of IMiDs on NK cells could be an important mechanism for enhancing anti-myeloma immune activity.

Lenalidomide treatment upregulates CD16, CD40L, and LFA1 in NK cells to promote antibody-dependent cytotoxicity (ADCC) and increases the number of CD56^dim^ NK cells as well as changes the ratio of CD56^bright/dim^ NK cell subpopulations (Tai et al., 2005; Fionda et al., 2018). Preclinical research revealed that after 2 weeks of lenalidomide treatment, an NK cell subpopulation with overexpression of CD56 and downregulation of NKp30, NKp46, and KIR2D emerged. Notably, this subpopulation disappeared after 4 weeks of therapy (Le Roy et al., 2018). How this dynamic change is specifically related to the therapeutic response remains unknown. Combined with the development and maturation of NK cells, this subgroup may be the primary group that exerts immunomodulatory effects during treatment. In addition, lenalidomide decreases PD-1 in NK cells and PD-L1 in MM cells, promoting NK cell identification of target cells (Giuliani et al., 2017). Lenalidomide can also activate Zap-70 triggering the phosphorylation and upregulation of GZM-B in NK cells (Hideshima et al., 2021). Furthermore, lenalidomide modulates the nanoscale rearrangement of actin in the immune synaptic cortex of NK cells. Subsequently, porous regions of the actin-network increase, promoting the release of interferon (IFN)-γ-containing vesicles. In summary, lenalidomide alters the immune phenotype via many pathways, adjusts the ratio of NK cell subpopulations, promotes the secretion of GZM-B and IFN-γ, and directly enhances the cytotoxicity of NK cells (Figure 3).

IMiDs indirectly promote the proliferation and activation of NK cells by regulating the complex signaling pathways of effector cells such as T cells, NK cells, and natural killer T (NKT) cells to promote IL-2 and IFN-γ release (Figure 3). The possible mechanisms are as follows: (a) IMiDs release inhibition of the IL-2 promoter in T cells via the CRBN–crl4–IKZF1/3–c-MYC/IRF4 pathway (Gandhi et al., 2014; Awwad et al., 2018; Asatsuma-Okumura et al., 2019; Zhu et al., 2019); (b) IMiDs stimulate T cells by CD28–NF-κB pathway (LeBlanc et al., 2004); (c) IMiDs promote the nuclear translocation of activated nuclear factor-2 and activator protein-1 by activating PI3K- PKCζ/Akt pathway in T cells (Hayashi et al., 2005); and (d) IMiDs activate the IL-2/IFN-γ-dependent JAK–Stat pathway after downregulation of suppressor of cytokine signaling 1 in effector cells (CD4/8 + T, NK, and NKT cells) (Görgün et al., 2010; Bhutani et al., 2019). Taken together, NK cells are the key effector cells of IMiDs. In patients with NDMM treated with lenalidomide and dexamethasone, those with low NK cell/Treg ratios had a significantly shorter PFS (19.8 months *versus* 57.3 months) than those with high ratios (Kim et al., 2022). The mature NK cell population of patients receiving maintenance therapy with IMiDs after transplantation exhibited a significant imbalance between activating and inhibiting receptors (NKG2D^+^Tim3^+^KIR2DS4^–^KIR3DL1^–^) (Bhutani et al., 2019). Furthermore, KIR2DS4^+^ NK cells were persistently elevated in patients with minimal residual disease (MRD) positive (Bhutani et al., 2019). Therefore, NK cells are a reasonable predictor of PFS during IMiD treatment. However, lenalidomide does not exert an effect on NK cells after microenvironmental immunosuppression or heavy pretreatment. According to a clinical trial (NCT01191060), chemotherapy or transplantation typically depletes mature NK cells, biasing NK cell lines toward an immature state. Longitudinal immunoassays were performed 1 month after completing lenalidomide monotherapy maintenance. No changes in NK cell counts were observed, and maturation status was independent of lenalidomide maintenance; furthermore, no improvement in depleted ADCC was noted (Besson et al., 2018). Next-generation immunomodulators are currently in the clinical trial phase. In MM settings, novel cereblon E3 ligase modulators (CELMoDs) in development include iberdomide (IBER) and mezigdomide (CC-92480). IBER increases the number of NK cells (NCT02773030) (Amatangelo et al., 2019). Mezigdomide activates NK cells and can induce proliferative NK cell populations even at concentrations 100-fold lower than pomalidomide. This immune activation capacity is not antagonized when combined with bortezomib (Bjorklund et al., 2021). A promising induction/maintenance therapy could be the exploration of more potent NK cell activation pathways to assist CELMoDs to coactivate the innate immunity of patients.

### 3.3 Basis of daratumum (Dara) efficacy: Functional NK cells

Dara is an anti-CD38 mAb. It exerts antimyeloma activity via antibody-dependent cell phagocytosis, ADCC, complement-dependent cytotoxicity, and immunomodulatory effects. The median time from DARA treatment to DARA-refractory recurrence (T0) in patients with NDMM was 50.1 months. The subsequent median OS (mOS) time from T0 was 8.6 months, 9.3 months for patients who received at least one follow-up treatment, and only 1.3 months for those who did not receive further treatment (Gandhi et al., 2019). This clearly indicates that disease progression was accelerated after Dara resistance and that patients eventually developed resistance-related disease relapse and died of MM.

NK cells, the major effector cells of mAbs, exhibit CD38 expression, second only to myeloma cells. After Dara monotherapy (SIRIUS and GEN501), CD38^+^ NK cells were decreased in a rapid, reversible, dose-dependent manner, dominated by the depletion of CD56^bright^ NK cells (Casneuf et al., 2021). However, NK cell populations recovered to approximately 50% within 3 months after the end of treatment (Casneuf et al., 2017). The remaining NK cells exhibited increased expression of CD69, CD127, CD25, CD27, and CD137 and decreased expression of CD45RA and GZM-B (Adams et al., 2019). Increased CD27 expression represents a higher cytolytic potential (Bullock, 2017). Decreased CD45RA expression is associated with immature CD56^bright^ NK cells (Krzywinska et al., 2016). Moreover, increased CD69, CD25, and CD137 expression is associated with an activated NK cell stage (Sabry et al., 2019). These findings suggest that persistent NK cells are in an activated state with cytotoxic potential but remain immature. Wang et al. have reported that Dara induced CD38^+^ NK cell fratricide via the ADCC effects of NK–NK cells, which is the main mechanism underlying NK cell reduction during treatment (Figure 3). CD38^−/low^ NK cells are selected via this mechanism. Compared to CD38^+^ NK cells, CD38^-/low^ NK cells had increased mitochondrial respiratory capacity, glycolytic rate, and glycolytic reserve, as well as compensatory transcriptomic features favoring OXPHOS metabolism and cholesterol synthesis (Naeimi Kararoudi et al., 2020; Woan et al., 2021). Increased levels of cytotoxic genes, such as IFNG and GzmB, were seen in iPSC-derived CD38 knockout (CD38KO) NK cells (Nagai et al., 2019). The aforementioned alterations suggest an increase in the metabolic activity of CD38^-/low^ NK cells, consistent with a rise in cytotoxicity. In addition, CD38^-/low^ NK cells resisted cell death induced by oxidative stress by increasing cysteine-glutathione disulfide synthesis, resulting in increased *in vivo* persistence (Cichocki et al., 2019; Woan et al., 2021).

Adaptive NK cells (KLRC2^hi^ FCrγ^−^) with low CD38 expression in patients with NDMM are effective in killing tumor cells in the presence of Dara (Cho et al., 2021). Patients with HRMM have a significantly lower proportion of adaptive NK cells, possibly explaining the poor response of this subpopulation to Dara (Cho et al., 2021). Therefore, CD38^−/low^ NK cells may be an outcome indicator for predicting Dara efficacy. Relatively recent preclinical studies have confirmed this hypothesis. Combination therapy of amplified NK cells with Dara was applied to MM tumor models. The anti-myeloma effect of untreated amplified NK cells was limited compared with that of CD38^−/low^ NK cells. Such cells may still be eliminated by Dara-mediated cellular self-mutilation (Table 1). Furthermore, CD38^+^ NK cells are rapidly eliminated but still relevant. Dara activates CD38^+^ NK cells, thereby inducing monocytes to increase the expression of T cell adaptor molecules (CD86/80) and differentiate into M1 macrophages with antitumor activity. This may represent the initial activation of the immune system by mAbs (Viola et al., 2021). The selection of CD38^−/low^ NK cells, potential immunomodulatory activity, and multiple mechanisms of killing myeloma cells explain why the direct effect of Dara treatment is not associated with a decrease in NK cell numbers in the Casneuf et al. study (Casneuf et al., 2017). However, multiple lines of attack may result in immune system exhaustion, and the recovery of depleted NK cells becomes challenging. This severely affects the efficacy of subsequent mAbs, results in treatment-related adverse effects and drug tolerance. Nahi et al. have reported that 39% of patients with progressive MM developed viral reactivation and infection-related complications during the period of decreased NK cell counts after Dara treatment (Nahi et al., 2019). In addition, the remaining myeloma cells during Dara treatment have low CD38 expression. They are not only not easily detectable but also not eliminated by mAbs and are a potential threat to disease relapse. Even when CD38 expression levels are restored, drug-resistant patients do not respond to DARA retreatment due to NK cell exhaustion (Nijhof et al., 2015). Recent studies have demonstrated in more detail that both primary (inadequate response to monotherapy) and acquired (disease progression after prior response) resistance to Dara is associated with NK cell dysfunction, as evidenced by reduced expression of CD16 and granzyme B, and increased expression of TIM-3 and HLA-DR (Verkleij et al., 2023). NK cells from healthy donors partially reversed drug resistance. Gene-edited NK cells with CD3^−/low^ CD16F158V and CD38KO-NK cells can target and eliminate CD38^−/low^ myeloma cells (Nagai et al., 2019; Sarkar et al., 2020). This may be because NK cells contain another set of germline-encoded receptor recognition mechanisms. Therefore, focusing on NK cells may prolong the timeline of patient resistance to mAbs and provide effective therapeutic approaches after resistance.

**TABLE 1:** A summary of typical preclinical studies regarding the combination of Dara and expanded NK cells for MM.

NK cells source	NK cells modification	Cells	Animals	Treatment	Result cells animals		Ref.
PB–NK cells of healthy donors	Experimental: Knock out CD38 (CD38^KO^ NK cells) Control: n-NK cells	MM.1S; H929; OPM-2; KMS-11, U266; DARA-resistant primary MM cells	NSG mice	Experimental: Dara + CD38^KO^ NK cells Control: Dara + n-NK cells	Higher ADCC(II)	No NK cells consumption	Naeimi Kararoudi et al. (2020)
Human iPSC	Experimental: express CD16a and IL-15/IL-15R (high affinity and non-cleavable; Knock out CD38 (iADAPT NK cells) Control: n-NK cells	MM.1R	NSG mice	Experimental: Dara + iADAPT NK cells	Higher Specific lysis (II)	Tumor load decreased by 89%	Woan et al. (2021)
				Control: Cell: Dara + n-NK cells			
				Mice: Dara			
PBMC of patients	No	U266; RPMI8226	NSG mice	Experimental: Dara + n-NK cells	Higher ADCC (I)	Experimental: All died on day 91	Thangaraj et al. (2021)
				Control: Dara		Control: All died on day 70	
PBMC of patients treated with Dara	CD38^-/low^ NK cells	MM.1S	NSG mice	Experimental: Dara+CD38^-/low^ NK cells	Higher Specific lysis (III)	Experimental: Survival rate:60% (day 60)	Wang et al. (2018)
				Control: Dara		Control: All died on day 50	
KHYG1 (aggressive NK cell leukemia patient)	CD38^-/low^CD16^F158V^ NK cells	JJN3; H929	—	Experimental: Dara+ CD38^-/low^CD16^F158V^NK cells	Higher IFN-γ and TNF-α release (H929: II JJN3: I)	—	Sarkar et al. (2020)
				Control: Dara+ CD38^-/low^ NK cells			
CMV-seropositive donors	FcεRIγ-negative NK cells (g-NK cells)	KMS11; KMS34; AM01; MM.1S; KMS18; LP1	NSG mice	Experimental: Dara + g-NK cells	Higher ADCC (II)	Experimental: Survival rate:100% (day 60)	Bigley et al. (2021)
				Control: Dara + n-NK cells		Control: All died on day 57	
PBMC of healthy donors	No	RPMI8226; U266	SCID	Experimental: Dara + n-NK cells	Higher Specific lysis (I)	Tumor volume decreased: Experimental: 6.6 times	Motais et al. (2021)
				Control: n-NK cells		Control:43 times	

Abbreviations: Dara: daratumumab; NK, cells: natural killer cells; PB: peripheral blood; NSG, mice: NOD. Cg-Prkdc^scid^Il2rg^tm1Wjl^/SzJ mice; n-NK, cells: normal NK, cells; BLI: bioluminescent imaging; ADCC: antibody-dependent cellular cytotoxicity; iPSC: induced pluripotent stem cell; PBMC: peripheral blood mononuclear cells; CMV: cytomegalovirus; I: Increase but less than 1 times (compared to the control group); II: More than 1 times (compared to the control group); III: More than 2 times (compared to the control group); SCID: severe combined immunodeficient mice.

### 3.4 NK cells are the main effector cell type of elotuzumab

The Food and Drug Administration (FDA) has approved elotuzumab in combination with lenalidomide and dexamethasone for the treatment of relapsed and refractory MM (RRMM) after 1–3 lines of treatment. SLAMF7-mediated myeloma killing depends on NK cell-mediated ADCC (Hsi et al., 2008). Conversely, SLAMF7 can activate NK cells directly via the EAT-2 signal pathway (Cruz-Munoz et al., 2009; Chen and Dong, 2019). In in vitro preclinical studies, the survival rate of NK cells co-cultured with elotuzumab was greater than 95% (Pazina et al., 2017). This was considerably different from the effect of Dara. Moreover, preclinical studies have reported that elotuzumab activates NK cells by directly binding to SLAMF7. SLAMF7-expressing NK cells do not die of fratricide. Instead, a unique activation pathway promotes SLAMF7–SLAMF7 interaction between NK and myeloma cells (Malaer and Mathew, 2017) (Figure 3). Compared with Dara, elotuzumab is more dependent on well-functioning NK cells. Because ADCC and NK cell activation are its primary antimyeloma mechanisms. When exploring the optimal sequence of the combination of the two mAbs, researchers observed that patients who first received Dara had a significantly lower response to elotuzumab compared with controls owing to the high depletion and slow recovery of NK cells (Hoylman et al., 2020). However, patients with RRMM who were treated with the combination of elotuzumab, carfilzomib, lenalidomide, and dexamethasone did not have activated NK cells. The highly immunosuppressive microenvironment of these patients may make NK cells hyporeactive and less prone to activation (Foureau et al., 2021). In such patients, no objective response was observed with elotuzumab monotherapy. Nevertheless, other drug combinations, such as PIs to sensitize myeloma cells or IMiDs to activate NK cell function, were effective in improving this nonresponsive state (Campbell et al., 2018). Preclinical research has partially explained the synergistic antimyeloma effect of elotuzumab and lenalidomide. According to this study, cross-talk between NK cells, monocytes, and myeloma cells increased adhesion between myeloma cells and NK cells by upregulating the adhesion molecule CD54 and stabilised the immune synapse (Richardson et al., 2023). During combination therapy with elotuzumab/IMiDs, the effective ratio of NK cells to myeloma cells was associated with a longer PFS and was a more accurate predictor of efficacy than cytogenetic HR status (Danhof et al., 2019). Elotuzumab/lenalidomide/amplified NK cells exhibited a very good partial response (VGPR) rate of 97% and an MRD-negative rate of 75% in patients who relapsed after transplantation (NCT01729091). Clinical trials have also validated the efficacy of combining amplified NK cells with elotuzumab in patients who did not achieve MRD negativity after transplantation, however, the results remain unreported (UMIN000033128). Elotuzumab can activate innate immunity. Therefore, exploring effective NK cell activation *in vivo* may restore the single-agent activity of elotuzumab and expand clinical use. Furthermore, exogenous NK cell infusion can help patients with RRMM maintain a good effector–target cell ratio and ensure the therapeutic efficacy of mAbs.

### 3.5 Recovery of NK cells after auto-HSCT is closely associated with efficacy maintenance

For patients who can undergo transplantation, auto-HSCT remains the first-line treatment option. However, recurrence and drug resistance after transplantation remain unavoidable. Furthermore, normal humoral and cellular immunity is restored after >1 year of transplantation. However, rapidly recovering NK cells can exert immune surveillance during this window. Therefore, several studies have comprehensively examined the association among NK cell counts, activation, specific immune reconstitution, and disease control after transplantation.

The higher the NK cell count, the longer the PFS, and it is also an independent predictor of MRD negativity after HSCT (Keruakous et al., 2022). After 2–3 months of HSCT, patients with normal NK cell counts have a 7.5-fold higher MRD-negative rate than those with low cell counts (Keruakous et al., 2022). Further, the relative balance of NK cell inhibitory or activating receptors may be an important factor for determining MRD status. Six years after auto-HSCT, researchers observed redistribution of NK cell inhibitory and activating receptors in patients with persistent CR, including decreased NKp46 and increased NKG2A and KIR2DL1 (Arteche-López et al., 2017). MRD-positive patients had fewer circulating NK cells following transplantation compared to MRD-negative patients. Circulating NK cells retained activation capacity, with upregulation of KIR2DS4 and downregulation of NKG2A (Bhutani et al., 2019). In a small clinical study (NCT02519114), whether stem cell transplantation with KIR ligand-mismatched NK cells can decrease the risk of myeloma recurrence was investigated. It was observed that patients relapsed within 90 days. However, post-transplantation cyclophosphamide treatment rapidly removed early mature graft-derived NK cells; therefore, the late reconstitution of functionally mature NK cells is responsible for the lack of response (Van Elssen et al., 2021).

During leukocyte recovery after transplantation, a distinctive pattern of immune reconstitution characterized by a markable decrease of CD56^dim^ NK cell and a marked expansion of CD56^bright^ NK cells was observed (Jacobs et al., 2015; Orrantia et al., 2021). The increased presence of CD56^bright^ NK cells was accompanied by elevated GZMB levels and upregulation of KIR2DL2/3/S2 and KIR3DL1 (Jacobs et al., 2015). In general, CD56^bright^ cells are immature NK cells that do not express CD57. However, during leukocyte recovery, the CD57^+^CD56^bright^ subset was expanded; furthermore, an immature NKG2A^+^CD57^−^ cell subpopulation was dominant in CD56^dim^ NK cells (Jacobs et al., 2015; Orrantia et al., 2021). NK cells undergo rapid division during leukocyte recovery. Genes related to biological processes such as cell cycle, DNA replication, and energy metabolism, including glycolysis and tricarboxylic acid cycle, were significantly enriched and returned to normal transcript levels after 1 month (Orrantia et al., 2022). Furthermore, during this period, decidual-like NK cells were expanded, characterized by CD9 expression. Compared with CD9^−^ NK cells, they had higher perforin and GZMB levels. However, the significance of the expansion of this subpopulation remains unverified (Orrantia et al., 2022). CD56^low^ CD16^low^ NK cells may represent an intermediate stage of differentiation and returns to peak at 2 weeks after transplantation, which is approximately the leukocyte recovery period (Vulpis et al., 2018). Taken together, the above changes suggest that NK cells recovered early after transplantation originate from immature cell populations rather than activated mature populations. However, during immune reconstitution, the cytotoxic potential and proliferation rate of these cells increase; this change may be owing to the reconstituted cytokine environment (Orrantia et al., 2021). After transplantation, adaptive NK cells are characterized by NKG2C^−^FCϵRγ^−^ (Orrantia et al., 2021). Patients with low NKG2C^hi^ NKG2A^low^ adaptive NK cell counts had more than two-fold higher recurrence rates than those with high counts (Merino et al., 2021). CD57^+^ NK cells tend to under-express the chemokine receptor CXCR4, affecting the homing of NK cells to the tumor primary site. At 30 and 100 days after auto-HSCT, patients who have a lower frequency of NKG2A^−^CD57^+^ NK cells have better PFS than those with a higher frequency. In summary, NK cells can identify and target residual myeloma cells during the critical window (first 3 months) of assessing MRD status. Combination therapies that activate NK cells before transplantation or combining NK cell infusions after transplantation can help restore immune surveillance and maintain a deeper remission state.

### 3.6 NK cells serve dual purposes as helper and effector cells in CAR therapy

CAR is a recombinant antigen receptor that facilitates antigen binding and effector cell activation. NK cells help maintain CAR-T cell function, as well as act as effector cells along with T cells. In March 2021, the FDA approved idecabtagene vicleucel (bb2121, targeting BCMA) for treating RRMM in adult patients after more than four treatment lines, including PIs, IMiDs, and anti-CD38 mAbs (Sharma et al., 2022). The favorable response rate of CAR-T cells in RRMM has been summed up in several reviews (Holstein et al., 2023; Parikh and Lonial, 2023; Zhang et al., 2023). However, the development of a sustained CAR-T cell response is still challenging, important potential processes include antigen loss, the generation of anti-CAR antibodies, and CAR-T cell exhaustion. Studies have indicated that 6%–8% of patients with MM undergoing BCMA CAR-T cell therapy experience antigen loss (NCT02215967; NCT02215967) (Ali et al., 2016; Brudno et al., 2018). These patients have a deletion in the BMCA gene and do not respond well to treatment (Da Vià et al., 2021; Samur et al., 2021). High levels of anti-CAR antibodies were produced in 7 out of the 17 RRMM patients treated with the bi-epitopic BCMA CAR-T (Cilta-cel). Six of them experienced relapses or progression within 6 months after infusion (Xu et al., 2019). Furthermore, a higher CD4:CD8 ratio and an increased frequency of CD45RO−CD27^+^CD8^+^ T cells were linked to patient responses to CAR-T cells, indicating that T-cell exhaustion is still a significant factor in efficacy (Garfall et al., 2019). Bachiller et al. combined CAR-T cell therapy with a low-dose infusion of expanded NK cells and revealed that NK cells promote early activation of CAR-T cells, enhance migration to tumor cells, and decrease the expression of the depletion markers PD-1, TIM3, and LAG-3 in CAR^+^ and CAR-T cells (Bachiller et al., 2021). T cell senescence affects the long-term persistence of CAR-T cells (Bluhm et al., 2018). Nevertheless, clinical trials of immune checkpoint inhibitors have been terminated owing to high toxicity (Castella et al., 2018). Therefore, NK cell infusion may be a promising strategy to improve the targeting and persistence of CAR-T cell.

Compared with CAR-T cells, CAR-NK cells are significantly less toxic and costly and do not present with graft-versus-host disease. They can recognize tumor cells via multiple mechanisms (natural receptors and CARs) to decrease off-target effects. Furthermore, in in vitro preclinical studies, the antitumor activity of CAR-NK cells was noted to be consistently higher than that of parental cells (Maroto-Martín et al., 2019). Compared with NKG2D CAR-T cells, NKG2D CAR-NK cells eliminated myeloma cells without targeting healthy cells in a mouse model (Leivas et al., 2018; Leivas et al., 2021). CD19 CAR-NK cells can target CD138^−^/CD19^+^ MM cells exhibiting some stem cell properties in in vitro preclinical studies (Zhao et al., 2018). On the other hand, CD38 CAR-NK cells efficiently lyse MM cells refractory to Dara without targeting other nonhematopoietic tissues expressing CD38 *in vitro*. Moreover, lysis efficiency is nonlinearly correlated with CD38 expression (Stikvoort et al., 2021). This nonlinear correlation may be a separate therapeutic benefit from NK cells. CXCR4–BCMA (dual targeting) -NK cells increase migration to the BM via the CXCR4–CXCL12/SDF-1α axis in a mouse model (Ng et al., 2022). Genetic engineering techniques can be employed for constructing CAR-NK cells. In in vivo preclinical studies, NK cells derived from induced pluripotent stem cells were genetically edited to exhibit the following characteristics: (a) expression of recombinant IL-15/IL-15 receptor signaling complexes; (b) expression of high-affinity, non-cleavable CD16; and (c) knockdown of CD38. Subsequently, these cells were transduced to the target CARs of BCMA (FT576) (Goodridge et al., 2020) and GPRC5D (FT555) (Reiser et al., 2022). These engineered cells do not require cytokine support during the expansion phase and provide continuous control of tumor growth when used alone; when combined with Dara, they clear myeloma cells. Preclinical studies on CAR-NK cells against other targets such as CD138 (Wei et al., 2018), and SLAMF7 are currently ongoing (Wang et al., 2020).

At present, three CAR-NK cell trials (NCT05008536, NCT03940833, and NCT05182073) are registered at ClinicalTrials.gov. In all these trials, BCMA was the target. These trials, which are currently in Phase I/II, are focusing on BCMA. The safety and initial efficacy of the iPSC-derived BCMA-CAR-NK cell phase I trial (NCT05182073) have been reported (Huang et al., 2023). The results of the remaining clinical trials are awaited. Compared with CAR-T cell-related clinical trials (149 enrollments and 8 completions), this approach is still in its infancy. Common challenges with expanded NK cell infusion remain, including low CAR transduction efficiency, poor *in vivo* persistence, and the need for multiple doses to ensure efficacy. Previous studies have reported that the BM microenvironment of patients with RRMM inhibits NK cell function. Therefore, we cannot ignore the possibility that CAR-NK cell activity is impaired at the tumor primary site. The use of nanobody-based CARs can be a promising approach for transducing highly soluble and stable CAR-NK cells (Hambach et al., 2020). This can be overcome in the future via effective NK cell activation or using advanced genetic engineering techniques that encode genes favoring sustained expansion.

### 3.7 BsAbs or trispecific antibodies targeting NK cells

BsAbs and trispecific antibodies simultaneously target effector cells (T/NK cells) and tumor cells to generate immune synapses, resulting in effector cell activation and tumor cell destruction. At present, 28 related clinical trials have been registered, and one trial has been completed (NCT00938626). Furthermore, there are ongoing preclinical investigations focused on the activation of NK cells. The novel NKG2D ligand–antibody fusion construct (ULBP2-BB4) improves specific cell lysis in in vivo and *in vitro* studies (von Strandmann et al., 2006). AFM26 targets BCMA and CD16A and induces effective lysis of primary myeloma cells *in vitro*, independent of CD16A polymorphism and not limited by the low copy number of BCMA (Ross et al., 2018). CS1-NKG2D BsAb activates the NKG2D–DAP10 complex on NK cells, thereby activating the phosphorylation of AKT to induce IFN-γ production and specific lysis of myeloma cells and significantly prolonging survival in mice (Chan et al., 2018). Moreover, CTX-8573/4,419 targets BCMA and NKp30 and promotes the lysis of NK against myeloma cells and exhibits potent antitumor efficacy *in vitro* and *in vivo*, with a broad therapeutic window (Draghi et al., 2019; Watkins-Yoon et al., 2019). 2A9-MICA efficiently recruited NK cells to specifically target tumor tissue and induced IFN-γ and tumor necrosis factor-α release in mice (Wang et al., 2020). NKG2D-2B4 BsAb promotes IFN-γ production to induce direct cytotoxicity and may be used in clinical settings to assess the functional activity of NK cells (Song et al., 2021). These BsAbs are the first to combine BCMA-targeted therapy with the NKG2D–NKG2DL axis. Nevertheless, there are no ongoing relevant clinical studies.

### 3.8 Adjuvant NK cells are essential for the functioning of DC vaccines

DC vaccine-based immunotherapy is in the clinical research stage. In total, 14 related clinical trials have been registered, 9 of which have been completed (Clinical Trials. gov). However, the clinical efficacy of these trials is limited (Verheye et al., 2022). Preclinical studies have investigated the role of NK cells in DC activation. CD83^+^CCR7^+^CD56^−^ NK cells can activate DCs as immunomodulatory helper cells (Mailliard et al., 2005). Furthermore, NK cells induce the maturation of Th1-polarized DCs, provide antigenic substances, and maintain cytotoxic activity against immature DCs. Reciprocally, DCs can facilitate cytokine production and the proliferation and cytotoxicity of NK cells (Van Elssen et al., 2014). In addition, the cytotoxic action of NK cells on immature DCs can prevent the interaction between immature DCs and T cells, ensuring the activation of adaptive immune responses. NK cell heterogeneity in different MM stages may lead to differences in DC vaccine efficacy. Therefore, drugs that activate NK cells, such as IMiDs, can improve the antitumor immune activity of vaccines, as demonstrated by Nguyen-Pham. The group treated mouse models with lenalidomide and DC vaccines, and observed that the proportion of activated NK cells was significantly higher, as was the tumor-suppressing effect (Nguyen-Pham et al., 2015). Therefore, treatment with DC vaccines and lenalidomide may produce a synergistic NK cell activation signal that positively correlates with tumor control.

### 3.9 HDACis bidirectionally regulate NK cell activity

The FDA has approved the use of panobinostat, an oral HDACi, in combination with bortezomib and dexamethasone for patients who have received more than two treatments, including bortezomib and IMiDs. In in vitro studies, HDACi can hamper the growth of lenalidomide-resistant MM cell lines by upregulating NKG2D ligands to enhance the ADCC effect (Hirano et al., 2021). Valproic acid-treated myeloma cells exhibit increased sensitivity to NK cell lysis owing to the upregulation of NKG2D ligands, which is caused by a more active ERK signaling pathway (Wu et al., 2012). However, HDACis significantly inhibit immune monitoring of NK cells by inhibiting activating receptors such as NKG2D, NKp44, NKp46, and CD25 and promoting cell apoptosis (Ogbomo et al., 2007; Rossi et al., 2012). Fiegler et al. have reported that HDACis downregulated the expression of NKp30 ligand B7-H6 and decreased the recognition of NKP30-dependent tumor cells (Fiegler et al., 2013). Therefore, similar to bortezomib, HDACis enhance NK cell-mediated lysis and negatively regulate cellular activity.

### 3.10 Unique NK cell subsets are an efficacy indicator of selinexor

The FDA has approved selinexor, a selective XPO1 inhibitor, for RRMM. In Fisher et al. ‘s study, lymphoma cells pretreated with selinexor exhibited markedly decreased HLA-E expression and increased sensitivity to NK cell-mediated killing (Fisher et al., 2021). Furthermore, they reported that increased numbers of immature CD56^bright^ subpopulations of patients with colorectal cancer treated with selinexor are associated with inferior treatment response (Fisher et al., 2021). Moreover, ABCC4 as a biomarker for predicting the treatment response and prognosis of patients with MM who received selinexor was significantly positively correlated with NK cell infiltration and TIM3 expression (Hu et al., 2022). ABCC4 belongs to the ATP binding box transporter family (Wen et al., 2015) and plays an important role in clinical multidrug resistance via drug efflux from tumor cells. NK cells may be associated with the clinical response to selinexor. Identifying the relevant predictors of selinexor efficacy may involve focusing on NK cell subsets associated with tumour control.

### 3.11 Venetoclax combined with NK cell gives patients with t (11; 14) individual treatment

Members of the B-cell lymphoma (BCL)-2 family regulate the apoptotic mechanism of myeloma cells in a stringent manner. Plasma cells of the CCDN1 subset carrying the t (11; 14) translocation express BCL-2 abundantly and are dependent on BCL-2 for survival (Lernoux et al., 2021). Venetoclax is the first FDA-approved BCL-2 inhibitor. In patients with RRMM carrying the t (11; 14), both monotherapy and combination therapy with venetoclax showed promising results Venetoclax monotherapy resulted in an ORR of 40% and a VGPR of 27% (NCT01794520); in combination with dexamethasone, that resulted in an ORR of 60% and a VGPR of 30% (NCT01794520) (Parrondo et al., 2022).

After treatment with venetoclax of healthy donor NK cells, NK cells with high expression of BCL-XL and MCL-1 were not inhibited in proliferation, exhibited upregulated NKG2D, elevated degranulation levels, and increased cytolytic toxicity. This indicates that venetoclax has the potential to act in concert with NK cells. In MM cell lines carrying the t (11; 14) and with elevated levels of CD38 and BCL-2, venetoclax combined with Dara increased ADCC activity (Nakamura et al., 2021). Venetoclax promotes apoptosis by activating caspase via the mitochondrial apoptotic pathway (Roca-Portoles et al., 2020). When NK cells exert ADCC, granzyme entry into cells also activates caspase via the mitochondrial apoptotic pathway (Prager and Watzl, 2019). Consequently, mitochondria-driven apoptosis may be the mechanism by which venetoclax and NK cells exert their synergistic effects. The combination of venetoclax and NK cells is expected to induce apoptosis at low concentrations and maintain efficient tumor cell destruction while overcoming the toxicity of venetoclax (Narni-Mancinelli and Vivier, 2022; Pan et al., 2022). Several studies are actively investigating NK cells with increased venetoclax resistance. For instance, NK cells can be stimulated by feeder cells expressing mbIL-21 (Yano et al., 2022). Alternately, the BCL2 G101V mutation was driven out of induced pluripotent stem cells (iPSCs) using the CRISPR-Cas9 system, and iPSCs with the BCL2G101V isotype were selected for differentiation into NK cells (Bernareggi et al., 2022). The edited NK cells were 94 times more resistant to venetoclax than their wild-type counterparts (Bernareggi et al., 2022). The combination of safe concentrations of venetoclax and NK cell is anticipated to result in superior individualized therapy for patients with t (11; 14) MM in the future.

## 4 Applications of NK cell therapy

NK cells are important in anticancer immunity. The strategies for restoring NK cells include endogenous recovery and exogenous infusion. Endogenous recovery includes cytokine activation and immune checkpoint suppression.

### 4.1 Cytokine-based activation

Cytokines are crucial for NK cell proliferation and activation. Patient-derived NK cells did not kill autologous myeloma cells. However, after the stimulation of the IL-2/15, they acquired an activated phenotype, with upregulation of NKp30, CD57, and TRAIL receptors, and regained lysogenic capacity (Tognarelli et al., 2018). Furthermore, NKG2A is the only inhibitory receptor that is upregulated upon cytokine stimulation. NKG2A blockade along with cytokine stimulation further increases the cytotoxicity of NK cells (Tognarelli et al., 2018). In in vitro and *in vivo* experiments, recombinant human IL-15 stimulation significantly increased the NKG2D^+^ NK cell population (Fernandez et al., 2023). In clinical trials (NCT01572493), IL-15 expanded CD56^dim^ and CD56^bright^ cell populations, enhanced the cytotoxicity of CD56^dim^ NK cells, accelerated the maturation of CD56^bright^ NK cells (Dubois et al., 2017). Moreover, *in vitro* preclinical studies have demonstrated that IL-15 alone activates NK cells with a short-lived advantage in tumor control and that co-activation with IL-12/15/18 helps to generate NK cells with memory properties that may contribute to a long-lasting antitumor effect (Bonanni et al., 2019). In this premise, inhibition of the C-X-C motif chemokine receptor 3 (CXCR3)/ligand axis increased the infiltration ability of IL-15-activated NK cells in the BM, inducing a strong and long-lasting antitumor effect in a mouse model (Bonanni et al., 2019). NKTR-255, an IL-15 receptor agonist that shifts the phenotypic balance of NK cells towards the activated phenotype, inhibited MM cells *in vitro* and *in vivo* when combined with Dara (Fernandez et al., 2023). Presently, IL-15 is widely used for NK cell activation (Table 2). A key limitation of NK cell immunotherapy is the inability of activated/expanded NK cells to enter the tumor site. Different stimulation regimens may differentially modulate the antitumor function of NK cells by affecting their tissue-homing properties. Cytokine-mediated endogenous NK cell activation can support traditional therapies to improve patient outcomes. Therefore, cytokine pretreatment of infused NK cells can be a potential strategy for cellular immunotherapy (Table 2).

**TABLE 2:** Summary of clinical trials on NK cell infusion (Data obtained from ClinicalTrials.gov). Abbreviations: auto-exp-NK cells: Autologous Expanded NK cells; MM: Multiple myeloma; ENK cells: Expanded Natural Killer; ASCT: Autologous Stem Cell Transplant; BMT: Blood and Marrow Transplant.

NCT number	Phases	Enrollment	NK cells	Combination	Conditions	Status	Title	Results first posted	Last update posted
NCT01884688	2	3	auto-exp-NK cells	\	Asymptomatic MM	Completed	UARK 2013-05 A Study of Autologous Expanded Natural Killer Cell Therapy for Asymptomatic Multiple Myeloma	2017.04	2017.04
NCT01313897	2	10	auto-exp-NK cells	Bortezomib	MM	Completed	UARK 2010-35, A Study of Expanded Natural Killer Cell Therapy for Multiple Myeloma	2017.04	2017.05
NCT03003728	2	0	ENK cells	Elotuzumab\ASCT\ ALT-803	MM	Withdrawn	2015-10: Expanded Natural Killer Cells and Elotuzumab for High-Risk Myeloma Post- Autologous Stem Cell Transplant (ASCT)	\	2020.07
NCT01040026	1\ 2	10	Allogeneic Expanded Haploidentical NK Cells	\	MM	Unknown status	Expanded Natural Killer (NK) Cells for Multiple Myeloma Study	\	2019.11
NCT02955550	1	15	PNK-007	rhIL-2	MM	Completed	A Safety Study of Human Cord Blood Derived, Culture-expanded, Natural Killer Cell (PNK-007) Infusion with or Without Subcutaneous Recombinant Human Interleukin-2 (rhIL-2) Following Autologous Stem Cell Transplant for Multiple Myeloma (MM)		2020.07
NCT02481934	1	6	auto-exp-NK cells	Lenalidomide\ Lenalidomide	MM	Completed	Clinical Trial of Expanded and Activated Autologous NK Cells to Treat Multiple Myeloma	2016.12	2016.12
NCT04558853	1	12	auto-exp-NK cells	\	MM	Active, not recruiting	Clinical Study of Autologous Natural Killer Cells in Multiple Myeloma	\	2021.02
NCT03019666	1	24	Nicotinamide Expanded Haploidentical or Mismatched Related Donor NK cells	\	MM	Recruiting	Ph I Trial of NAM NK Cells and IL-2 for Adult Pts with MM and NHL	\	2021.08
NCT04309084	1	29	CYNK-001	\	MM/Plasmacytoma	Active, not recruiting	Natural Killer Cell (CYNK-001) Infusions in Adults with Multiple Myeloma	\	2022.05
NCT00185757	1	20	NK cells	cytokine	MM/BMT	Unknown status	Cytokine Induced Killer Cells as Post-Transplant Immunotherapy Following Allogeneic Hematopoietic Cell Transplantation	\	2012.12
NCT00720785	1	35	auto-exp-NK cells	Bortezomib	MM	Completed	Natural Killer Cells and Bortezomib to Treat Cancer	\	2022.06
NCT04558931	2	60	auto-exp-NK cells	Isatuximb	MM	Recruiting	Clinical Trial for Autologus NK Cells Alone or in Combination with Isatuximab as Maintenance for Multiple Myeloma	\	2022.03
NCT05400122	1	12	NK Cells	IL-2\ TGF-β receptor I inhibitor	MM	Not yet recruiting	Natural Killer (NK) Cells in Combination with Interleukin-2 (IL-2) and Transforming Growth Factor Beta (TGFbeta) Receptor I Inhibitor Vactosertib in Cancer	\	2022.06
NCT02890758	1	14	NK cells	ALT803	MM	Active, not recruiting	Phase I Trial of Universal Donor NK Cell Therapy in Combination with ALT803	\	2021.07
NCT00477035	1	22	auto-exp-NK cells	cytokine	MM	Completed	Post-transplant Autologous Cytokine-induced Killer (CIK) Cells for Treatment of High Risk Hematologic Malignancies	\	2017.01
NCT05008536	1	27	Anti-BCMA CAR-NK Cells	Fludarabine\ Cytoxan	MM, Refractory	Recruiting	Anti-BCMA CAR-NK Cell Therapy for the Relapsed or Refractory Multiple Myeloma	\	2021.11
NCT03940833	1\2	20	BCMA CAR-NK 92 cells	\	MM	Unknown status	Clinical Research of Adoptive BCMA CAR-NK Cells on Relapse/Refractory MM	\	2019.05
NCT05182073	1	168	FT576 (Allogenic CAR NK cells with BCMA expression)	Daratumumab	MM	Recruiting	FT576 in Subjects with Multiple Myeloma	\	2022.07

### 4.2 Potential functions of immune checkpoint inhibitor

The balance between the activating and inhibitory receptors on the NK cell surface regulates the recognition and killing of target cells. Therefore, inhibitory receptors targeting NK cells may restore of immune surveillance *in vivo*. In MM, combination therapy with lenalidomide and anti-KIR mAb 1–7F9 (IPH2101) increased NK cell-associated tumor clearance (Benson et al., 2011). However, heterogeneity in KIR expression can hinder mAbs targeting KIRs, which results in limited therapeutic responses (NCT01248455 and NCT01217203). NK cell depletion is associated with high levels of TIM3 in solid tumors. The anti-TIM3 antibody can improve cellular function (Gallois et al., 2014). The interaction between TIM3 and its ligand galectin-9 induces NK cell-mediated IFN-γ production, increasing IDO1 levels in tumor cells to maintain immune escape (Folgiero et al., 2015). Presently, the efficacy of anti-TIM3 antibodies remains unclear as relevant studies regarding MM microenvironments are lacking. NK cells from NDMM or RRMM present moderate levels of TIGIT (Guillerey et al., 2018). Whether NK cells with high TIGIT levels promote or inhibit myeloma growth is controversial. In a preclinical study, the anti-TIGIT antibody was more effective than the control and anti-PD-1 antibody in reducing myeloma burden and prolonging mice survival (Guillerey et al., 2018). However, a previous study has reported that TIGIT on NK cells promoted cellular function and may be a tumor protective factor for acute myeloid leukemia (Jia et al., 2018). TIGIT and DNAM-1 share a common ligand, the poliovirus receptor (Mekhloufi et al., 2020). DNAM-1 is a crucial activation receptor for NK cells. High TIGIT levels may competitively inhibit DNAM-1 activation, leading to the “anergic” state of NK cells. Unknown is how elevated TIGIT levels affect NK cell function and the immune microenvironment in MM. Recent cellular experiments demonstrate that EZH2 inhibitors augment the antitumor effects of TIGIT monoclonal antibodies by modulating the TIGIT-CD155 axis between NK and MM cells (Liu et al., 2023). Reportedly, NK cells in patients with advanced MM express PD-1, and anti-PD-1 therapy can increase the targeted lysis of MM cells. However, single-agent clinical trials (NCT01222286/NCT00999830) have reported poor efficacy. In RRMM, a combination regimen including anti-PD-1 antibody and IMiDs was suspended because of its adverse effects. Recently, Susek et al. reported the discovery of novel NK cell specific PD1-based chimeric switch receptors (PD1-CSR), in which transduced NK cells enhance and maintain potent antitumor activity in the PD-L1+ microenvironment (Susek et al., 2023). A clinical trial of BCMA–PD1–CAR-T cells (NCT04162119) is ongoing; however, the efficacy is yet to be determined. LAG3 is expressed on the surface of activated and mature NK cells and is a negative regulator of cytokine production (Narayanan et al., 2020; Chen et al., 2023). Understudied in MM is the inhibition of LAG3 on the surface of NK cells. Inhibitory receptors may act differently in different microenvironments. As a result, strategies for the safe and effective use of immune checkpoint inhibitors in MM remain unexplored. Lanuza et al. proposed NK cell adoptive transfer as a novel strategy to overcome the abovementioned inhibitory pathways. In particular, KIR ligand-mismatched allogeneic NK cells may remain unaffected by the immunosuppressive effects of host tumor cells (Fiegler et al., 2013; Lanuza et al., 2019).

### 4.3 Potential of expanded NK cell infusion

In contrast to the uncertain clinical benefits of cytokine activation and immune checkpoint inhibition, preclinical and clinical studies have provided promising results for NK cell adoptive transfer.

In in vitro preclinical studies, cord blood-derived expanded NK cells effectively lysed primary myeloma cells and could be used alone, without mAbs, against MM (Reina-Ortiz et al., 2020). NK cells depleted of the NKG2A-encoding gene exhibited significant cytotoxicity against all myeloma cell types (Bexte et al., 2022). KIR ligand-mismatched NK cell from healthy donors downregulated inhibitory signaling pathways and increased ADCC (Mahaweni et al., 2018). The edited KHYG-1 NK cell line from patients with aggressive NK cell leukemia presented CD16F158V^+^CD38^low^ characteristics. Subsequent, these cells can eliminate Dara-refractory myeloma cells *in vitro* (Sarkar et al., 2020). Amplified CD38^−/low^ NK cells derived from Dara-treated patients proliferated efficiently *in vitro*, exhibited increased lytic toxicity against MM.1S cells, and significantly improved the survival of mice (Wang et al., 2018). Expanded FcεRIγ^−^ NK cells from cytomegalovirus-seropositive donors effectively targeted various MM cell lines and sustained tumor growth control, with 100% survival in mice up to the observed endpoint (Bigley et al., 2021).

PNK-007 is a CD56^+^/CD3^−^ NK cell product expanded from placental CD34^+^ cells. In a phase II clinical trial (NCT02955550), the MRD-negative rate increased from 26% to 66% after PNK-007 infusion (Holstein et al., 2019). Another study (NCT01729091) enrolled 30 patients with HRMM who received expanded autologous NK cell infusion (cord blood-derived) after auto-HSCT. The distribution of VGPR and MRD-negative rates among these patients increased by 24% and 35%, respectively. (Srour et al., 2022). Six patients were the infused with expanded autologous NK (NKAE) cells after auto-HSCT (NCT04558853). New subsets (NKG2D^hi^, 2B4^hi^, TIM3^hi^, TIGIT^hi^, and CD38^low\−^) appeared within 4 h after infusion. Among the three patients with a VGPR before infusion, one maintained the original state, one relapsed in the fifth month, and one achieved CR (Nahi et al., 2022). Five patients with RRMM who underwent 2–7 lines of therapy received multiple infusions of NKAE cells (NCT02481934). Four patients showed disease stabilization, two patients showed a 50% decrease in BM infiltration, and one patient experienced a long-term response (Leivas et al., 2016). Patients who achieve an MRD-negative status after transplantation or chemotherapy have the most favorable effector–target cell ratios compared with those with higher tumor loads. To date, most clinical studies on expanded NK cell infusion for MM are still in phase I and II (Table 2), and effective expansion and maintenance *in vivo* remain unresolved. In clinical trials, cytokine activation therapy was administered before infusion (Table 2) to maintain cell expansion and function. The combination of expanded NK cell infusion and endogenous activation remains a promising therapeutic strategy for the future.

Individual genetic disparities among patients have been shown to be associated with NK cell sensitivity. PFS is influenced by personalized gene composition associated with the threshold of NK cell function, as demonstrated in clinical studies (NCT01749969). KIR3DL2+HLA−A3/11+ and the high-affinity FCGR3A-158V allele promoted ADCC, whereas KIR2DL1+HLA−C2C2+ inhibited ADCC (Sun et al., 2021). Recent CRISPR-based single-cell analyses revealed that the interaction between NK and myeloma cells induced distinct transcriptional activation states. Myeloma-intrinsic genes that control NK cell sensitivity and resistance have been identified. Myeloma cells with NLRC5 mutations and overexpression of selected genes including TNFRSF10D, NCR3LG1, ULBP1, PVR, and PCGF5 were sensitive to NK cells. Myeloma cells with TRAF3 and WHSC1 mutations and overexpression of TNFRSF10D, NCR3LG1, ULBP1, PVR, and PCGF5 were tolerance to NK cells. To create optimal NK cell-based therapies for myeloma patients, it is necessary to take individual genetic differences into account (Dufva et al., 2022).

## 5 Conclusion

In MM, post-treatment drug resistance is a pressing challenge to overcome. NK cells play a key role in immunosurveillance and targeted killing of tumor cells and are potential effector cells for existing therapies. Comprehensive knowledge of NK cell responses that facilitate disease control during the treatment, including PIs, IMiDs, and mAbs, is warranted. Optimal utilization of these responses may help overcome treatment-related drug resistance. NK cells are present throughout MM development. NK cell-based combination therapy may benefit patients with HR or RRMM and holds promise for achieving long-term MRD negativity. Additionally, the existing staging system can no longer meet the requirements of risk prediction; therefore, the establishment of a treatment response-based dynamic and accurate risk prediction model is an important prerequisite for developing individualized treatment in the future. Because the flexibility of NK cells varies with different treatments, they are an important component of this model that may be developed in the future. However, studies reporting the association between NK cells and clinical staging and risk stratification are lacking, and additional studies regarding the same are necessary. At present, how to effectively promote NK cell recovery is a hot topic of research. Finally, the development of genetic engineering technologies may lead to safer and more effective NK cell-related therapeutic strategies.
